# Dual Effects of miR-181b-2-3p/SOX21 Interaction on Microglia and Neural Stem Cells after Gamma Irradiation

**DOI:** 10.3390/cells12040649

**Published:** 2023-02-17

**Authors:** Hong Wang, Zhao-Wu Ma, Feng-Ming Ho, Gautam Sethi, Feng Ru Tang

**Affiliations:** 1 Radiation Physiology Lab, Singapore Nuclear Research and Safety Initiative, National University of Singapore, Singapore 138602, Singapore; 2 The School of Basic Medicine, Health Science Center, Yangtze University, 1 Nanhuan Road, Jingzhou 434023, China; 3 Department of Pharmacology, Yong Loo Lin School of Medicine, National University of Singapore, Singapore 117600, Singapore

**Keywords:** γ-irradiation, microglia activation, neurogenesis, miR-181b-2-3p, SOX21

## Abstract

Ionizing radiation induces brain inflammation and the impairment of neurogenesis by activating microglia and inducing apoptosis in neurogenic zones. However, the causal relationship between microglial activation and the impairment of neurogenesis as well as the relevant molecular mechanisms involved in microRNA (miR) remain unknown. In the present study, we employed immunohistochemistry and real-time RT-PCR to study the microglial activation and miRNA expression in mouse brains. Real-time RT-PCR, western blot, ELISA, cell proliferation and cytotoxicity assay were used in BV2 and mouse neural stem cells (NSCs). In the mouse model, we found the acute activation of microglia at 1 day and an increased number of microglial cells at 1, 7 and 120 days after irradiation at postnatal day 3 (P3), day 10 (P10) and day 21 (P21), respectively. In cell models, the activation of BV2, a type of microglial cell line, was observed after gamma irradiation. Real-time RT-PCR analysis revealed a deceased expression of miR-181b-2-3p and an increased expression of its target SRY-related high-mobility group box transcription factor 21 (SOX21) in a dose- and time-dependent fashion. The results of the luciferase reporter assay confirmed that SOX21 was the target of miR-181b-2-3p. Furthermore, SOX21 knockdown by siRNA inhibited the activation of microglia, thereby suggesting that the direct interaction of 181b-2-3p with SOX21 might be involved in radiation-induced microglial activation and proliferation. Interestingly, the gamma irradiation of NSCs increased miR-181b-2-3p expression but decreased SOX21 mRNA, which was the opposite of irradiation-induced expression in BV2 cells. As irradiation reduced the viability and proliferation of NSCs, whereas the overexpression of SOX21 restored the impaired cell viability and promoted the proliferation of NSCs, the findings suggest that the radiation-induced interaction of miR-181b-2-3p with SOX21 may play dual roles in microglia and NSCs, respectively, leading to the impairment of brain neurogenesis.

## 1. Introduction

MicroRNAs (miRNAs) can act as potential biomarkers for different neurological as well as neuropsychological disorders and radiation exposure [1,2]. For instance, our recent report demonstrated that miRNA-34a-5p is involved in the impairment of neurogenesis and the development of depression in adult mice after early life irradiation [3]. The miR-181 family (miR-181a/b/c/d), which is highly evolutionarily conserved, has been involved in inflammation, apoptosis, ischemia and stroke [4,5,6]. The downregulation of miR-181-a/b/c in the rat brain is observed after transient focal ischemia [7]. In the ischemic penumbra of mouse models, miR-181-a/b/c/d expression decreased, but after transient focal ischemia, the expression increased in the ischemic core. The injury in a mouse stroke model was exacerbated by miR-181a [8], which induced neuro-2a cell death [9], the dysfunction of mitochondria and the increase in the cell death of astrocytes through modulating the family members of B cell lymphoma (Bcl)-2 [8]. In contrast, miR-181c suppressed Toll-like receptor 4 expression and downstream cytokines and protected the neurons [10,11]. In another study, Ma et al. demonstrated that miR-181c contributed to brain injury in acute ischemic stroke by promoting the apoptosis of microglia and neurons via the modulation of pro- and anti-apoptotic proteins [6]. Ye et al. reported that the miR-181/PKC-δ pathway was involved in microglial activation and the deterioration of neurodegeneration [12]. miR-181b has been identified as one of the most dysregulated microRNA during the course of chronic lymphocytic leukemia, and it has been implicated in inflammation by regulating the NF-κB signaling pathway. In addition, irradiation induced miR181 in the human lymphoblast cell line TK6 [13]. Sun et al. also suggested that miR-181b served as a potent regulator of downstream NF-κB signaling in the vascular endothelium and could provide a new target for anti-inflammatory therapy and critical illness [14].

Given that miR-181b-2-3p is downregulated by doxorubicin (DOX), which may be involved in DOX-induced toxicity [15], and that curcumol, an anti-inflammatory antioxidant, upregulated miR-181b-2-3p [16], we investigated whether radiation induced changes in miR-181b-2-3p and its target gene as well as protein in the mouse brain and in vitro microglial and neural stem cell models in order to understand its role in radiation-induced neurotoxicity, including brain inflammation and the impairment of neurogenesis. The results may provide clues for designing novel therapies by targeting miR-181b-2-3p to produce radio-neuro-protective effects. 

SRY-related high-mobility group box (HMGB) transcription factor 21 (SOX21) belongs to the Sox transcription factor family. Sox1, Sox2 and Sox3 are expressed by most progenitor cells during the development of CNS and are reported to suppress neurogenesis by maintaining neural cells in an undifferentiated state [17,18]. On the contrary, SOX21 was demonstrated to promote neuronal neurogenesis by counteracting the activity of Sox1, Sox2 and Sox3 in the chick embryo [19] and in the mouse hippocampus [20]. However, no evidence has been published regarding the role of SOX21 in microglial activation, which has been demonstrated to exhibit cross-talk with CNS progenitor cells during neurogenesis [21]. The inhibition of microglial activation was demonstrated to improve adult neurogenesis in the rat hippocampus during sleep deprivation [22]. The prediction of miR-181b-2-3p targets using TargetScan showed that SOX21 was one of the main targets of miR-181b-2-3p. We therefore hypothesized that SOX21, as a target of miR-181b-2-3p, played a key role in γ-irradiation-induced microglial activation and the impairment of neurogenesis. The present study systematically examined the number of and morphological changes in microglia in the mouse dentate gyrus at different days, i.e., 1, 7 and 120 days after γ-irradiation at postnatal day 3 (P3), P10 and P21. The effects of irradiation on microglia activation and neurogenesis, and the roles of the miR-181b-2-3p and SOX21 involved, were investigated in BV2 cell and neural stem cell models. 

## 2. Materials and Methods

### 2.1. Animal Models

Balb/c mice were housed in the Animal Care Facility at the National University of Singapore. The animals were provided food and water under a temperature of 22 °C and a 12 h light/12 h dark cycle. The Institutional Animal Care & Use Committee (IACUC), National University of Singapore, approved the experimental procedure (IACUC Number: R15-1576). 

The mice at postnatal days 3 (P3), 10 (P10) and 21 (P21) received whole body irradiation with 5 Gy (dose rate: 3.33 Gy/min) inside a BIOBEAM GM 8000 Gamma-Irradiator. The samples were collected at 1, 7 and 120 days post-irradiation for different experimental studies.

### 2.2. Immunohistochemical Staining

The animals were anesthetized at 1, 7 and 120 days post-irradiation with a mixture of medetomidine (1 mg/kg) and ketamine (75 mg/kg) at 0.1 mL/10g and perfused with 4% paraformaldehyde. The mouse brain was dissected, postfixed overnight, and transferred to 30% sucrose. The sagittal brain sections were cut at 40 μm. The sections were incubated with 3% H_2_O_2_ for 10 min, followed by goat serum, ionized calcium-binding adapter molecule 1 (IBA1) antibody (1:4000; Abcam, Cambridge, UK) overnight, secondary goat anti-rabbit antibody for 1 h, avidin–biotin complex (ABC) and DAB Substrate (Vector Laboratories Inc., Burlingame, CA, USA). The microscope (Leica Microsystems GmbH, Wetzlar, Germany) was used to take images. IBA1 immunopositive cells in the granule cell layer and hilus of the dentate gyrus were unbiasedly analyzed using the Stereologer System (SRC Biosciences Inc., Tampa, FL, USA). The results were indicated as the number/volume (mm^3^).

### 2.3. Mouse Brain RNA Extraction 

The brain was removed from the skull and cut into left and right halves along the midline. The cerebrum was collected and kept at −80 °C for further use. The Qiagen miRNeasy Mini Kit was used to extract RNA. The left-side cerebrum was homogenized in Qiazol lysis reagent, and RNA extraction was performed according to the manufacturer’s instructions. All of the purified RNA was eluted in RNase-free water and stored at −80 °C. The RNA concentration and integrity were checked by Nanodrop before being subjected to qRT-PCR analysis.

### 2.4. Real-Time RT-PCR Analysis for miR-181b-2-3p

For the cDNA synthesis of miRNA, miScript II RT kit (Qiagen, Hilden, Germany) was used to reversely transcribe RNA. For each 20 µL reaction, 5 µL template RNA was added to a 15 µL master mix comprising 4 µL 5× HiSpec buffer, 2 µL 10× nucleotide mix, 2 µL reverse transcript mix and 7 µL nuclease-free water. The procedure (37 °C for 1 h followed by 95 °C for 5 min) was used. A total of 80 µL of nuclease-free water was added into the resulting cDNA.

A master mix of 20 µL for real-time PCR contained: 10 µL 2× miScript SYBR green PCR master mix, 2 µL diluted cDNA, 2 µL primer for target miRNA and 2 µL 10× miScript universal primer, and 4 µL nuclease-free water. The following primers were used: 5′-CTC ACT GAT CAA TGA ATG CAA A-3′ for miR-181b-2-3p; 5′-AAT TCG TCA CTA CCA CTG AGA-3′ for Snor234; 5′-CTCGCTTCGGCAGCACA-3′ for U6. A Real-Time PCR System (Thermo Fisher Scientific, Waltham, MA, USA) was used to carry out PCR amplification. The samples were denatured at 95 °C for 15 min, followed by 40 cycles of reaction (denaturation at 94 °C for 15 s, annealing at 55 °C for 30 s and extension at 70 °C for 30 s). The fluorescence data were collected after the extension step. Snor234 was used as an internal control for the expression of miR-181b-2-3p in the mouse brain and BV2 cells, while U6 was used for NSCs.

### 2.5. Predication of miR-181b-2-3p Targets and Luciferase Reporter Assay

The online database TargetScan was employed to analyze the potential target genes of miR-181b-2-3p. SOX21, one of the miR-181b-2-3p targets, was selected for further analysis due to its involvement in neuroinflammation. To validate the direct interaction of SOX21 with miR-181b-2-3p, a luciferase reporter assay was carried out according to the previous manuscript [23]. The Mouse Sox21 3′-UTR region containing the miR-181b-2-3p binding sequence was amplified by RT-PCR using the primers 5′-TGG TGT TTG CTT TGC ACT TC-3′ and 5′-ATT CTG TGC TTT CTT TCT GTC CT-3′. The amplified fragments were cloned into the psiCHECK-2 plasmid (Promega Corporation, Waltham, WI, USA), with the PmeI and XhoI unique restriction enzyme sites located at the Renilla luciferase gene downstream. The firefly luciferase was used to normalize the transfection efficiency.

The Phusion Site-Directed Mutagenesis Kit (Thermo Fisher Scientific, MA, USA) was used to mutate the miR-181b-2-3p seed region of SOX21 3′UTR by primer sequences of 5′-phosphate GACCTGACCAGCTCGAATCC-3′ and 5′-phosphate AGTGTCAAGAAGAGATCCGG-3′ for site 1 and 5′-phosphate CTG GAA CGT TGA GTC TTG GG-3′ and 5′-phosphate TCAGCATACACACATTTACAATC-3′ for site 2. 

HEK293T cells were co-transfected with 100 nM scrambled mimic control or miR-181 mimic (Dharmacon, Lafayette, Lafayette, CO, USA) and 0.2 μg psiCHECK-2 containing miR-181b-2-3p binding sites in SOX21 3′-UTR by siRNA Transfection Reagent (Roche, Basel, Switzerland). A total of 48 h after the transfection, luciferase and renilla signals were measured using the Dual-Luciferase^®^ Reporter Assay System (Promega Corporation, Madison, WI, USA).

### 2.6. Culture of BV2 and Neural Stem Cells (NSCs)

Mouse BV2 and cortical neural stem cells (NSCs) were bought from Elabscience Biotechnology Inc. (Houston, TX, USA) and R & D Systems, Inc. (Minneapolis, MN, USA), respectively. BV2 cells were cultured in DMEM with 5% FBS and passaged with 2.5% trypsin. All of the abovementioned reagents were purchased from Gibco, Thermo Fisher Scientific, MA, USA. 

For NSCs, Matrigel (Thermo Fisher Scientific, MA, USA) was used to pre-coat cell culture flasks or plates. The NSCs were cultured in NeuroCult™ basal medium and proliferation supplement (STEMCELL Technologies Pte Ltd., Singapore) containing basic fibroblast growth factor (bFGF) and epidermal growth factor (EGF) (Invitrogen, Waltham, MA, USA). 

### 2.7. Western Blot 

At designated time points after the exposure to different doses of gamma irradiation, the cells were collected in CelLytic Lysis Reagent (Sigma-Aldrich Corporation, St. Louis, MO, USA) with 100X Halt™ Phosphatase and Protease Inhibitor Cocktail (Thermo Fisher Scientific, MA, USA), and then they were incubated on ice for 20 min with violent vortex several times. The supernatant was collected after the centrifugation at 15,000× *g* for 15 min. The protein concentration was measured by the BCA Protein Assay Kit (Thermo Fisher Scientific, MA, USA). 

The protein samples were run on 10% SDS-PAGE gel and transferred to the nitrocellulose membrane. The membranes were blocked and incubated with primary antibodies (SOX21: 1:1000, Abcam, Cambridge, UK; β-actin and Translocator protein (TSPO): 1:1000, Cell Signaling Technology, Beverly, MA, USA) overnight at 4 °C and HRP-conjugated secondary antibodies (1:10,000, Santa Cruz Biotechnology, Inc. Dallas, TX, USA) at room temperature for 1 h. Immunoreactive proteins were then visualized by ECL Prime Western Blotting Detection Reagent from GE healthcare (Buckinghamshire, UK). The Bio-Rad Gel Doc system was used to capture and quantify the images. β-actin was used as the loading control. The band densities of the target proteins were normalized to the loading control, and the fold change to the respective control groups was calculated.

### 2.8. RNA Extraction from Cells and Real-Time RT-PCR Analysis for miRNA and mRNA

The RNA extraction from cells and the real-time RT-PCR analysis for miRNA were performed as described above.

For mRNA real-time RT-PCR analysis, a first-strand cDNA synthesis kit (Thermo Fisher Scientific, MA, USA) was used to reversely transcribe RNA. A total of 20 µL of the reaction mix contained 1 µg RNA, 2 µL Maxima Enzyme Mix and 4 µL 5X Reaction Mix. The procedure was 25 °C for 10 min, 50 °C for 45 min and 85 °C for 5 min. A total of 100 µL of nuclease-free water was added into the resulting cDNA.

A master mix of 20 µL for real-time PCR contained: 10 µL 2× Maxima SYBR Green qPCR master Mix, 2 µL diluted cDNA, 4 µL nuclease-free water and 2 µL 10× target gene forward and reverse primers. The following primers were used for GAPDH: 5′-GCACCGTCAAGGCTGAGAAC-3′ and 5′-TGGTGAAGACGCCAGTGGA-3′. The following primers were used for SOX21: 5′-GCC GGT GAC TCG TGT CTT TA-3′ and 5′-GAA CGG CGG TCA TCT CTC AT-3′.

A Real-Time PCR System (Thermo Fisher Scientific, MA, USA) was used to carry out PCR amplification. The samples were initially denatured at 95 °C for 10 min, followed by 40 cycles of: denaturation at 95 °C for 15 s, annealing at 60 °C for 30 s and extension at 72 °C for 30 s. The fluorescence data were collected after the extension step. The GAPDH gene was used as an internal control.

### 2.9. Knock-Down Gene Expression of SOX21 by SOX21 siRNA in BV2

On day 1, 1 × 10^5^ BV2 cells were seeded in a culture medium in a six-well plate. On day 2, the cell medium was changed to Gibco opti-MEM reduced-serum medium (Thermo Fisher Scientific, MA, USA) and incubated for 2 h. siGENOME mouse SOX21 siRNA or siGENOME non-targeting control siRNA (Dharmacon, Lafayette, CO, USA) was mixed with 4 µL X-tremeGENE siRNA Transfection Reagent (Sigma-Aldrich Corporation, USA) for 15 min at room temperature. The mixture was then added to the cells in the six-well plate. After incubation for 5 to 6 h, the opti-MEM reduced-serum medium was replaced with DMEM medium. On day 3, the BV2 cells were exposed to 5 Gy gamma irradiation. On day 4, the cells were harvested for experiments. 

### 2.10. TNF-a in BV2 by Enzyme-Linked Immunosorbent Assay (ELISA)

ELISA kits (R & D systems, MN, USA) were used to measure the levels of TNF-α in cell lysates. Capture antibody was used to pre-coat the 96-well plate. A total of 100 µL of the standard or sample was added into the 96-well plate and kept at room temperature for 2 h, followed by the reagent diluent, the respective detection antibodies and, then, the streptavidin-HRP and substrate. The microplate reader (Thermo Fisher Scientific, MA, USA) was used to measure the absorption at 450 nm. TNF-α levels were calculated based on the standard curve. 

### 2.11. Cell Proliferation and Cytotoxicity Assay in NSCs

NSCs (100 µL) were seeded in 96-well plates at 15,000 cells per well. After reaching 80 to 90% confluency, the cells were exposed to different doses of γ-irradiation. Non-irradiated cells were used as the control. 

For the cell proliferation assay at designated time points, 10 µL CCK-8 solution (Dojindo Molecular Technologies Inc., Rockville, MD, USA) was added into each well and incubated for 4 h in the incubator. The absorbance at 450 nm was measured using a microplate reader (BioTek, Winooski, VT, USA). The cell proliferation level was expressed as the optical density. 

The cytotoxicity on NSCs induced by γ-irradiation was examined by the lactate dehydrogenase (LDH) assay (Dojindo Molecular Technologies Inc., Rockville, MD, USA). At 24 h after the gamma irradiation with different doses, 10 µL of lysis buffer (considered as the high control) was added to five wells in the non-irradiated plate and incubated at 37 °C for 30 min in a 5% CO_2_ incubator. Another five wells without the lysis buffer in the non-irradiated plate were considered as the low control. The supernatant (100 µL) from each well was transferred to an optically clear 96-well plate, and 100 µL of the working solution was added. The plate was protected from light and incubated at room temperature for 30 min, and then 50 µL of the stop solution was added to each well. The absorbance at 490 nm was measured using a microplate reader (BioTek, Winooski, VT, USA). The cytotoxicity of the gamma irradiation was determined using the following equation: Cytotoxicity = (reading after gamma irradiation–low control)/(reading from high control–low control).

### 2.12. Overexpression of SOX21 in NSCs

Lipofectamine 3000 reagent (Thermo Fisher Scientific, MA, USA) was used to transfect NSCs with mammalian vector pCMV6-AC-GFP containing SOX21 (NM_177753) Mouse Tagged ORF Clone (No: MG223510) or blank control pCMV6-AC-GFP (No: PS100010, OriGene Technologies, Inc. Rockville, MD, USA), according to the manufacturer’s instructions.

### 2.13. Statistical Analysis

The Fisher–Pitman permutation test was used in this study. The analyses were performed by the permutation function in Python. *p* < 0.05 was considered as statistically significant. The data are shown on a dot plot, with the additional mean ± SEM presented. 

## 3. Results

### 3.1. Exposure to γ-Radiation Induced Microglial Activation and Proliferation in the Mouse Dentate Gyrus 

**Figure 1:**
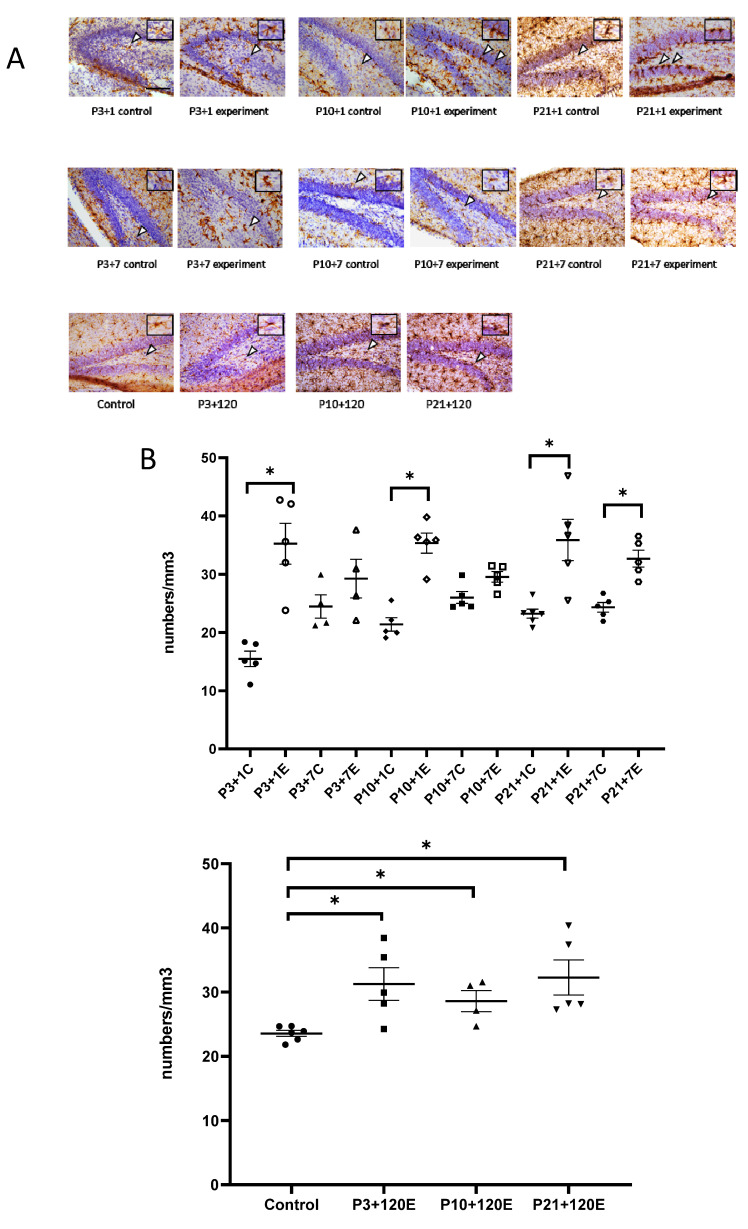

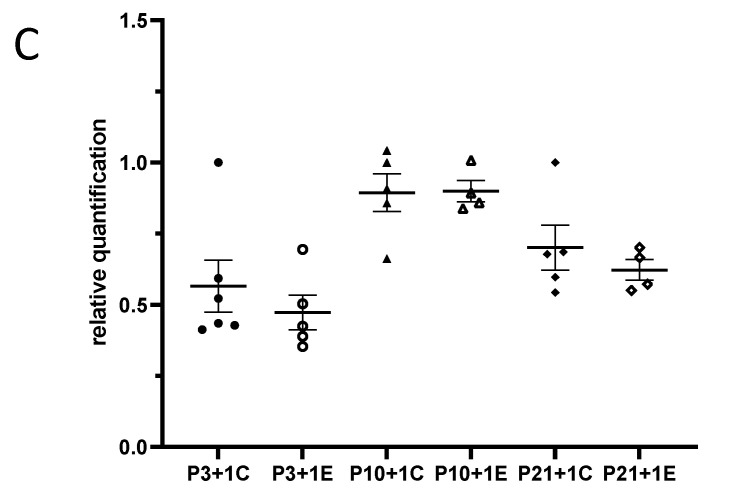
IBA1 immunostaining results indicated that γ-irradiation with 5 Gy at postnatal days 3, 10 and 21 induced the activation and increased the number of microglia in the dentate gyrus at 1 day and 120 days after radiation exposure when compared with the respective controls. At 7 days after irradiation, a significantly increased number of microglia occurred after irradiation at P21, but not at P3 and P10. (**A**): Immunohistochemical images of IBA1 staining in the dentate gyrus. Arrowheads show IBA1-positive immunostaining cells. Scale bar = 100 µm; Scale bar in the P3 + 1 control applies to other groups in this figure. (**B**): Statistical analysis of IBA1 immunopositive cell numbers (n = four to six). The data have been expressed as the mean ± SEM. * *p* < 0.05 vs. respective control. (**C**): The expression of miR-181b-2-3p in the mouse brain one day after γ-irradiation with 5 Gy at P3, P10 and P21 mice. There was no significant difference between the control and irradiated mice in the mouse brain. IBA1: ionized calcium binding adapter molecule 1.

In the developing brain, microglial precursors are amoeboid in morphology and progressively increase their ramifications into adulthood. In our study, amoeboid-shaped microglia were observed in the P4 (experimental at 1 day after irradiation at P3 and control P3 + 1) mouse brain. One day after irradiation at P3, Iba1-immunopositive microglia showed bigger cell bodies with thicker processes than those from the P3 + 1 control mice. Interestingly, the number of Iba1-immuno-positive microglial cells was increased in the granule cell layer and the hilus of the dentate gyrus (DG) one day after irradiation at P3 (Figure 1A,B). In P3 + 7 or P10 + 1 control mice, microglial cells had a ramified appearance, with a small cell body and multiple processes. Activated microglia showed a stout cell morphology at 7 days after irradiation at P3. Compared to the ramified microglia in the control mice, the number of reactive microglia appeared to be slightly increased, although not statistically significantly. At one day after irradiation at P10, the microglia showed darker and larger cell bodies with shorter and thicker processes. The activated microglia lined up along the subgranular zone (SGZ) and infiltrated into the stratum granulosum (granular layer) (Figure 1A). The increase in the number of microglia was statistically significant at 1 day but not at 7 days after irradiation at P10 when compared to the age-matched control mice (Figure 1B). One day after irradiation at P21, large and darkly stained Iba1-immunopositive microglial cells were observed in the SGZ of DG, and their thick processes protruded to the stratum granulosum, thus suggesting a dramatic microglial reaction to radiation exposure. On the contrary, the control mice showed ramified microglia with thin extensions and small round somas, whereas those in the irradiated group displayed shorter and thicker branches and larger cell bodies. Moreover, significantly increased reactive microglia were observed at 1 day and 7 days after irradiation at P21 and at 120 days after irradiation at P3, P10 and P21 (Figure 1A,B). Although remarkable changes in the microglia morphology were observed after γ-irradiation at a dose of 5 Gy, the qRT-PCR results showed that the expression of miR-181b-2-3p was not significantly different between the irradiated and control mice (Figure 1C).

### 3.2. SOX21 Was Highly Expressed in Microglia as the Target of miR-181b-2-3p

**Figure 2:**
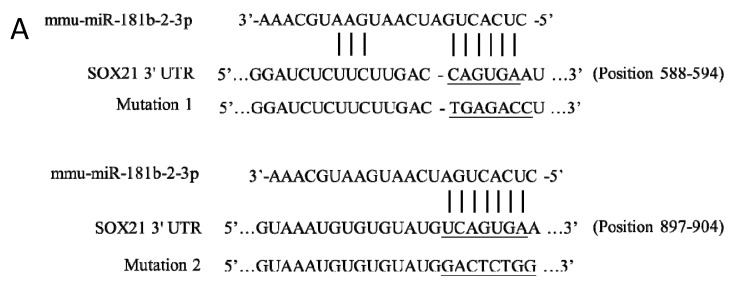

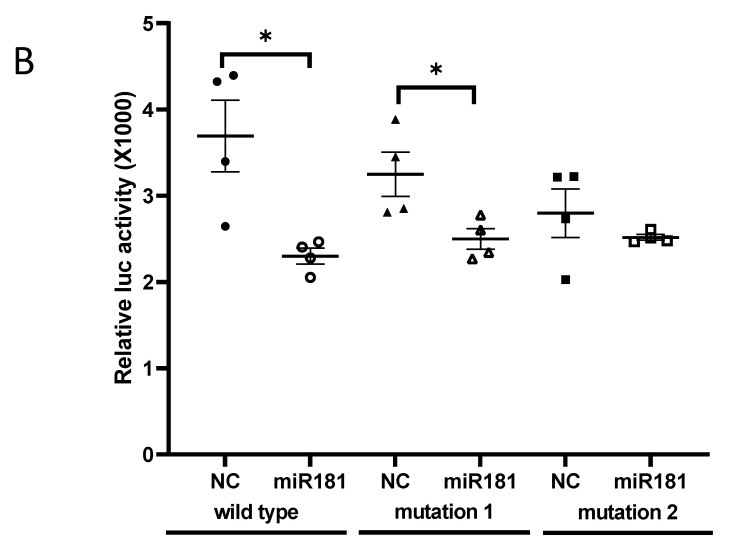
Direct interaction of miR-181b-2-3p with SOX21, as examined by the luciferase reporter assay. (**A**) Sequence alignment of two putative binding sites of miR-181b-2-3p in SOX21 3′-UTRs and the mutations. (**B**) Luciferase gene activity connected to SOX21 3′-UTR mRNA. HEK293T cells were co-transfected with scrambled mimic control or miR-181b-2-3p mimic and psiCHECK-2 constructed with 3′-UTR miR-181b-2-3p binding sites. A total of 48 h after transfection, the renilla and luciferase signals were measured. The data have been expressed as the mean ± SEM. * *p* < 0.05.

Two binding sites of mouse miR-181b-2-3p in SOX21 3′-UTR were predicted at positions 588–594 (site 1) and 897–904 (site 2) (Figure 2A). The direct interaction of site 2 with SOX21 was confirmed by the luciferase reporter assay. When miR-181b-2-3p mimic was co-transfected with psiCHECK-2 containing the mouse SOX21 3′-UTR binding sequence into HEK cells, the luciferase intensity decreased significantly compared to the negative control, thereby demonstrating the binding of miR-181b-2-3p and the 3′-UTR region of SOX21 (Figure 2B). We mutated site 1 and site 2 of SOX21 3′UTR, respectively. The cells still showed a significant decrease in luciferase activity after the transfection of SOX21 3′UTR with site 1 mutation, indicating that site 1 was not a real interaction binding site for miR-181b-2-3p (Figure 2B). Mutation 2 transfection inhibited the reduction in luciferase intensity, which indicated that miR-181b-2-3p could not bind to the mutant site 2 of SOX21 3′-UTR (Figure 2B). These results suggested that SOX21 was a direct target of miR-181b-2-3p.

### 3.3. Exposure to γ-Irradiation Significantly Decreased miR-181b-2-3p Expression and Increased the mRNA and Protein Levels of SOX21 in BV2 

**Figure 3:**
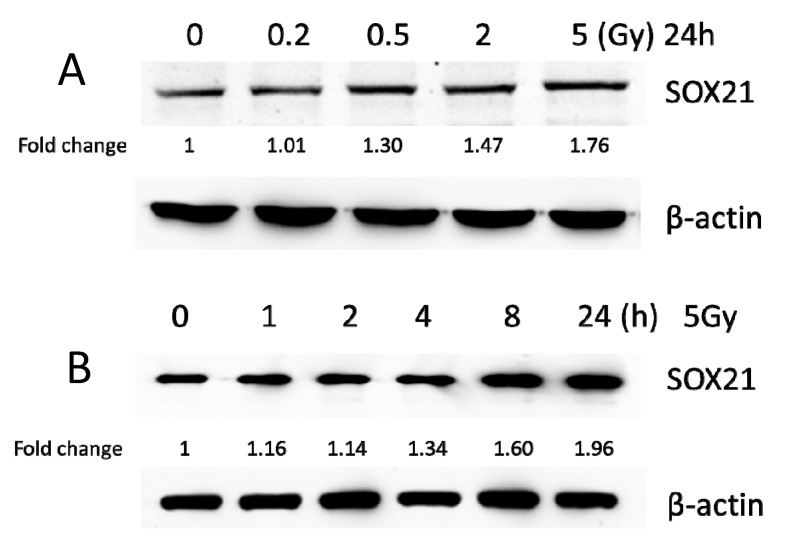

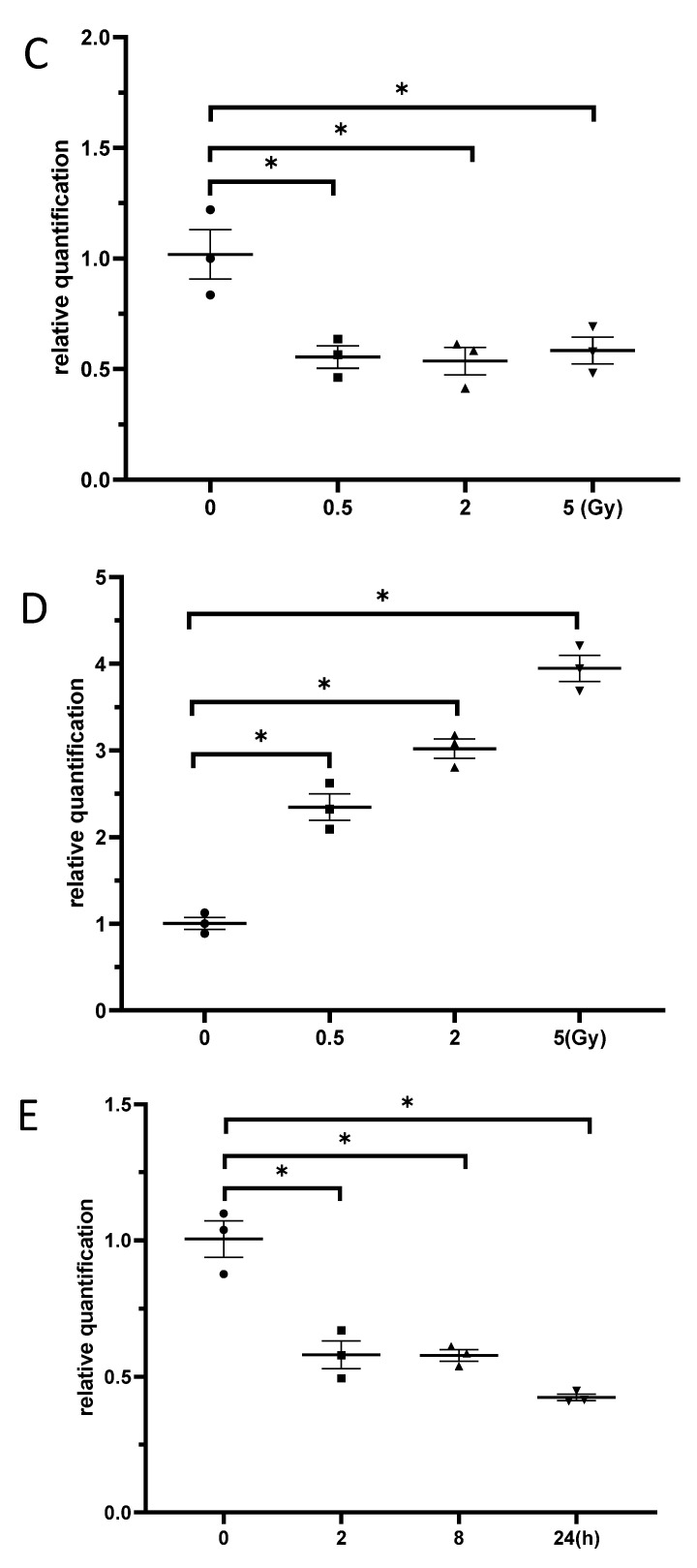

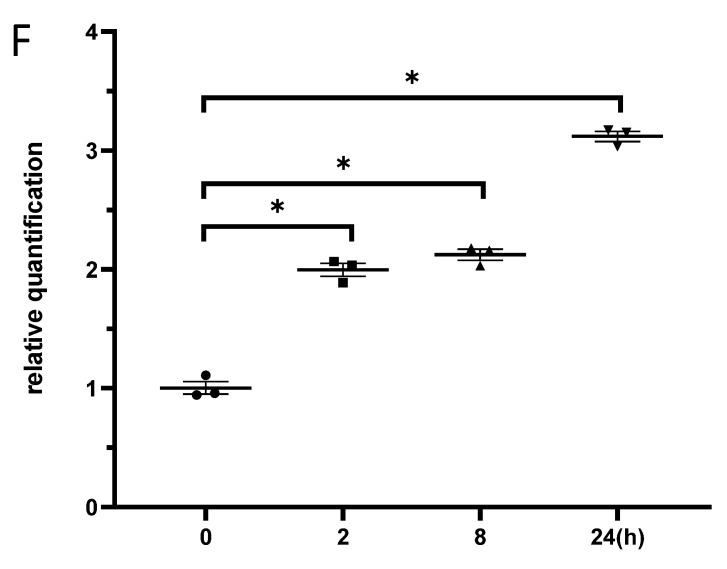
The expression of miR-181b-2-3p and SOX21 in BV2 cells after γ-irradiation. SOX21 protein expression in BV2 cells was evaluated by western blot in a dose- (**A**) and time-dependent (**B**) manner after γ-irradiation. Dose-dependent changes in miR-181b-2-3p (**C**) and SOX21 (**D**) mRNA expression in BV2 occurred 24 h after γ-irradiation. Time-dependent changes in miR-181b-2-3p (**E**) and SOX21 (**F**) mRNA expression in BV2 occurred after γ-irradiation with 5 Gy. The data have been expressed as the mean ± SEM (n = three). * *p* < 0.05 vs. control.

BV2 cells were exposed to γ-irradiation with diverse doses from 0.2 to 5 Gy. The results from the western blot indicated that the protein levels of SOX21 in BV2 increased in a dose-dependent manner after γ-irradiation (Figure 3A). Moreover, the protein levels of SOX21 time-dependently increased in BV2 cells after γ-irradiation with 5 Gy (Figure 3B). The qRT-PCR results indicated a dose-dependent decreased expression of miR-181b-2-3p from 0.5 to 5 Gy but an increased SOX21 mRNA expression after γ-irradiation (Figure 3C,D). The decreased expression of miR-181b-2-3p and the increased expression of SOX21 were also observed in BV2 from 2 h after exposure to 5 Gy γ-irradiation (Figure 3E,F). This negative correlation between miR-181b-2-3p and SOX21 mRNA expression further confirmed that SOX21 was a direct target of miR-181b-2-3p.

### 3.4. The Knockdown of SOX21 by SOX21 siRNA Blocked the Activation of Microglia Induced by γ-Irradiation

**Figure 4:**
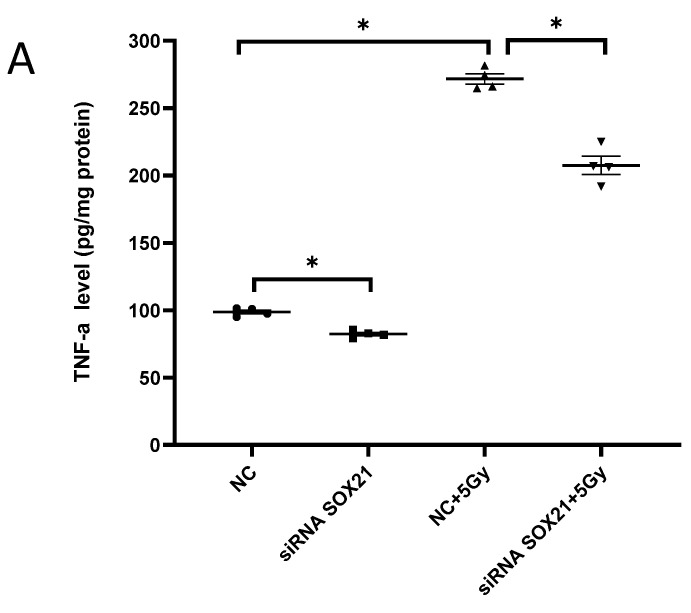

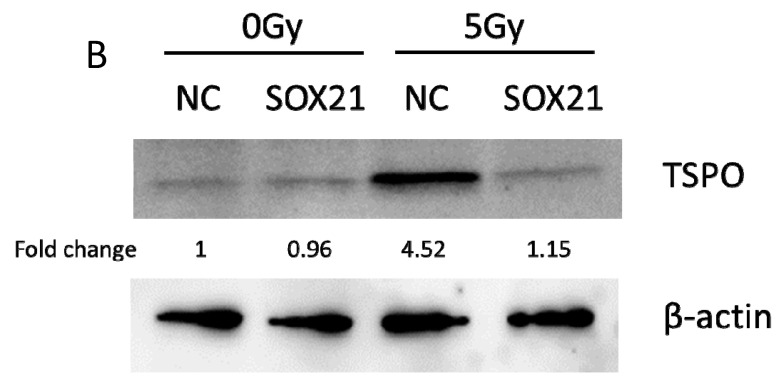
The activation of microglia by γ-irradiation was blocked by the knockdown of SOX21. The activation of microglia was evaluated by measuring the expression of TNF-α with ELISA (**A**) and of TSPO with western blot (**B**). TNF-α: tumor necrosis factor-α; TSPO: translocator protein. * *p* < 0.05.

The activation of microglia was further evaluated by analyzing the expression of TNF-α and TSPO. Exposure to γ-irradiation at 5 Gy induced the activation of BV2 cells, as indicated by the enhanced expression of TNF-α and TSPO in BV2 cells transfected by NC (Figure 4A,B). The upregulation of TNF-α induced by γ-irradiation was partially blocked upon the transfection of SOX21 siRNA, while SOX21 siRNA dramatically inhibited the increased TSPO protein level after γ-irradiation with 5 Gy (Figure 4A,B). These results indicated that the knockdown of SOX21 by SOX21 siRNA blocked the activation of microglia induced by γ-irradiation.

### 3.5. Exposure to γ-Irradiation Caused Cytotoxicity in NSCs and Induced the Impairment of Cell Proliferation

**Figure 5:**
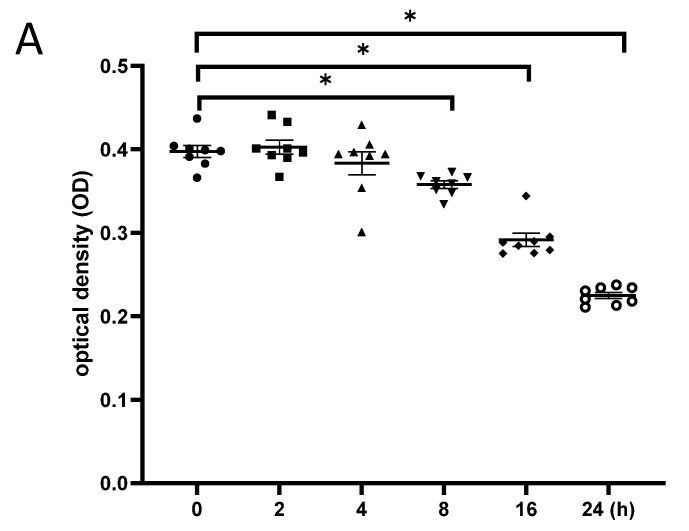

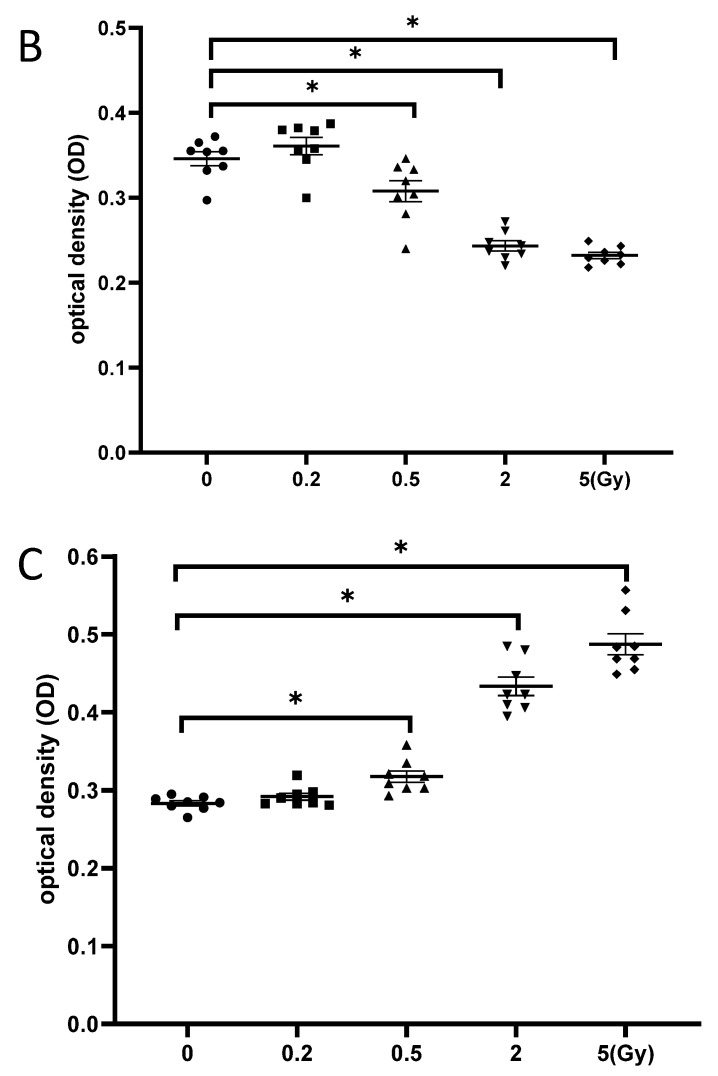
γ-irradiation reduced the proliferation of NSCs. (**A**): NSCs were irradiated with 5 Gy, and cell proliferation was evaluated at different time points after radiation exposure; (**B**): NSCs were irradiated with different doses, and cell proliferation was evaluated at 24 h post-irradiation; (**C**): Cytotoxicity at 24 h after irradiation with different doses of gamma irradiation. NSC: neural stem cells. The data have been expressed as the mean ± SEM. * *p* < 0.05 vs. control.

NSCs were γ-irradiated with 5 Gy, and cell proliferation was examined at different time points after irradiation. The CCK-8 test showed no significant change in cell proliferation until 4 h post-irradiation, while cell proliferation decreased from 8 to 24 h, thereby demonstrating the time-dependent effects of γ-irradiation on neurogenesis (Figure 5A). Moreover, NSCs exhibited a dose-dependent decrease in cell proliferation at 24 h after exposure to gamma irradiation, as significantly fewer cells were observed after irradiation with 5 Gy than with 0.5 Gy (Figure 5B). Additionally, the LDH assay indicated significantly increased cytotoxicity against NSCs at 24 h after exposure to 5 Gy γ-irradiation compared to that at 24 h after exposure to 0.5 Gy γ-irradiation (Figure 5C). This result was consistent with the dose-dependent impairment of neurogenesis caused by γ-irradiation.

### 3.6. Exposure to γ-Irradiation Increased the Expression of miR-181b-2-3p and Decreased the mRNA and Protein Expression of SOX21 in NSC 

**Figure 6:**
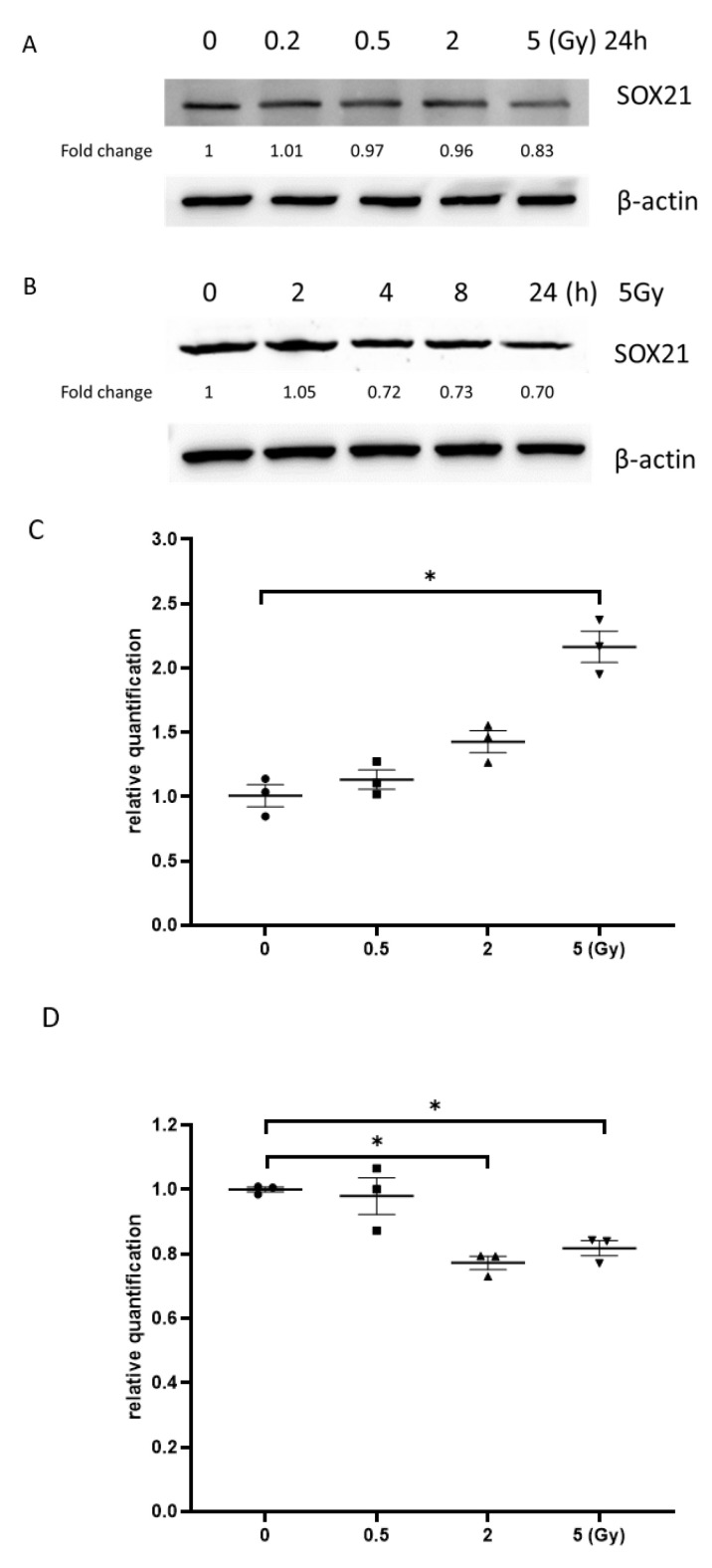

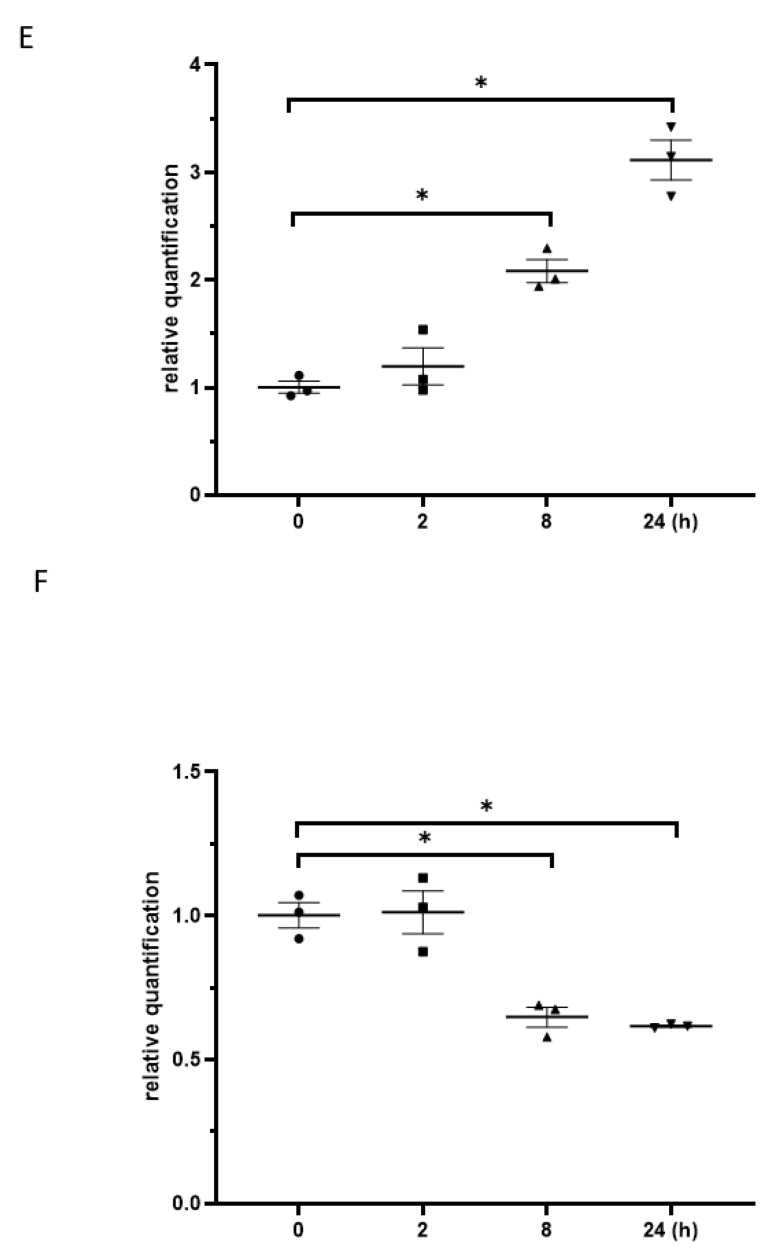
miR-181b-2-3p and SOX21 expression in NSCs after exposure to γ-irradiation. The decrease in SOX21 protein expression in NSCs, as evaluated by western blot, is dose- (**A**) and time-dependent (**B**) after γ-irradiation. Dose-dependent changes in miR-181b-2-3p (**C**) and SOX21 (**D**) mRNA expression in NSCs occurred 24 h after γ-irradiation. Time-dependent changes in miR-181b-2-3p (**E**) and SOX21 (**F**) mRNA expression in NSCs occurred after γ-irradiation at 5 Gy. NSC: neural stem cells. The data have been expressed as the mean ± SEM (n = 3). * *p* < 0.05 vs. control.

The results from the western blot exhibited that the exposure to γ-irradiation decreased SOX21 protein levels in NSCs dose-dependently (Figure 6A). When NSCs were exposed to γ-irradiation with 5 Gy, the reduction in SOX21 protein levels was found to be time-dependent (Figure 6B). The qRT-PCR results further indicated an increased expression of miR-181b-2-3p accompanied by the decreased mRNA expression of SOX21 in NSC after irradiation with 5 Gy (Figure 6C,D). The expression of SOX21 decreased at 8 and 24 h, but not at 2 h, after 5 Gy γ-irradiation in NSCs, whereas the expression of miR-181b-2-3p increased from 8 h in NSCs (Figure 6E,F). 

### 3.7. The Overexpression of SOX21 Blocked the Decreased Cell Viability Induced by γ-Irradiation in NSCs

**Figure 7:**
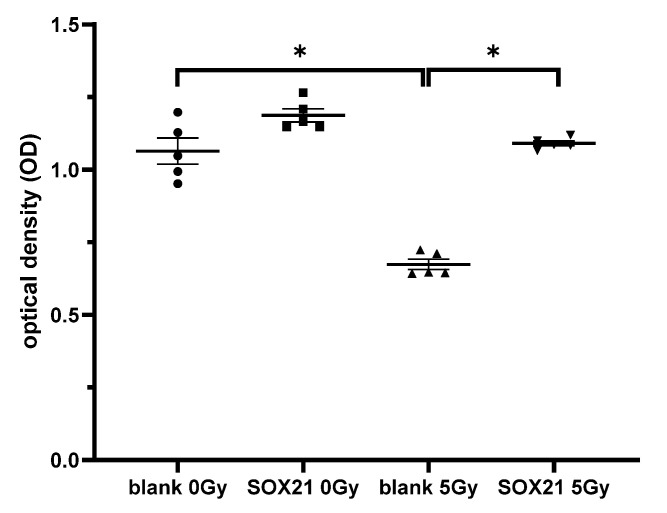
The effect of the overexpression of SOX21 in NSCs after γ-irradiation. NSCs were transfected with pCMV6-AC-GFP containing SOX21 or blank control and then γ-irradiated with 5 Gy as the experimental or 0 Gy as the control group. The cell proliferation in the transfected NSCs was evaluated by CCK-8. NSC: neural stem cells. The data have been expressed as the mean ± SEM (n = five). * *p* < 0.05.

The cell proliferation ability of NSCs was examined after the overexpression of SOX21 and γ-irradiation. When the cells were transfected with a blank plasmid, the cell proliferation decreased significantly after γ-irradiation with 5 Gy (Figure 7). This decrease was blocked upon the transfection of a plasmid containing the SOX21 sequence in NSCs (Figure 7), thereby suggesting that the overexpression of SOX21 might prevent γ-irradiation-induced detrimental effects.

## 4. Discussion

### 4.1. Irradiation-Induced Microglial Reaction Was Primarily Regulated by the Downregulation of miR-181b-2-3p with Upregulated SOX21

Microglial activation is associated with the incidence of brain injury induced by radiation exposure [24,25,26]. Activated microglia can release a diversity of proinflammatory cytokines and mediators, such as TNF-α, IL-1, ROS, NO, COX-2 and prostaglandin E [24,27], which are responsible for inflammation-related brain injury. Neuroinflammation can inhibit the proliferation of neural precursor cells and hippocampal neurogenesis, and an inflammatory blockade with anti-inflammatory drugs restores and augments neurogenesis after cranial irradiation [25,28]. Our recent study demonstrated that early life (i.e., at postnatal day 3) radiation exposure in mice caused abnormal brain development and the impairment of neurogenesis in the dentate gyrus, leading to adult depression [3]. The present study further systematically examined the microglial reaction in the dentate gyrus of mice after acute radiation exposure to 5 Gy at postnatal days 3, 10 and 21 and investigated the relevant molecular mechanisms. We showed activated microglia and an increased number of Iba1-positive microglial cells in the granule cell layer and the hilus of the dentate gyrus at 1, 7 and 120 days after gamma radiation exposure at different postnatal days. In the BV2 microglial cell model, we found that the interaction of downregulated miR-181b-2-3p with upregulated SOX21 may be related to radiation-induced microglial activation and inflammation in the dentate gyrus. High-dose γ-irradiation with 8 Gy caused the activation of microglia and increased the mRNA expression of cytokines in the human microglial cells CHEM5 [29]. An MRI scan in mice showed a significant brain region- and age-dependent radiosensitivity after early postnatal irradiation with 5 Gy [30]. The hippocampal region of P3 mice exposed to 5 Gy radiation exhibited significant reductions in volume. Acute irradiation reduced the dividing cells and newly generated neurons in the subgranular zone of the dentate gyrus, too [30].

miRNAs play important roles in the regulation of radiation-induced brain damage or protection [3,31,32]. miR-124 may act as a potential candidate in the mitigation of radiation-induced cognitive dysfunction, as the overexpression of miR-124 in mice reduced microglial activation in the irradiated brain and ameliorated the cognitive dysfunction [31]. On the contrary, the radiation-induced upregulation of miR-741-3p was involved in microglial activation, brain inflammation and neuronal apoptosis in the mouse hippocampus, as the downregulation of miR-741-3p reduced the number of GFAP-positive astrocytes and improved the protrusion and branching status of microglia after irradiation [32]. Furthermore, antagomiR-741 inhibited the levels of the proinflammatory cytokines IL-6 and TNF-α in the hippocampus. The delivery of an miR-741-3p inhibitor downregulated miR-741-3p expression and alleviated radiation-induced neuronal apoptosis and cognitive dysfunction at 6 weeks post-irradiation [32]. miRNA-181 is highly expressed in the brain, and miR-181c exacerbates brain injury in acute ischemic stroke by promoting the apoptosis of neurons [6]. miR-181a/b/c/d time-dependently decreased in BV2 cells during LPS-induced microglial activation, while the transfection of miR-181a/b/c/d mimics attenuated microglial activation in BV2, accompanied by the downregulation of iNOS, NO and ROS [12]. It is consistent with our current results that the expression of miR-181b-2-3p decreased time- and dose-dependently in BV2 cells after the exposure to gamma irradiation. A previous study suggested that the downregulation of miR-181b-2-3p may be toxic [15], whereas antioxidants upregulate miR-181b-2-3p [16]. In this study, the radiation-induced downregulation of miR-181b-2-3p may be related to its interaction with SOX21, a protein that regulates cell differentiation, leading to microglia activation, proliferation and subsequent brain inflammation and damage. The interaction of miR-181b-2-3p and its target SOX21 was confirmed by a luciferase array. The expression of SOX21 increased markedly in BV2 after the exposure to gamma irradiation. Thus far, no study has been published regarding the involvement of SOX21 in regulating microglia function. The microglia activation, which was indicated by the higher levels of TNF-α and TSPO, was observed in BV2 after γ-irradiation with 5 Gy. The transfection of SOX21 siRNA significantly blocked this increase, thus demonstrating that the knockdown of SOX21 expression could inhibit the activation of microglia.

### 4.2. The Irradiation-Induced Impairment of Neurogenesis Was Regulated by the Upregulated miR-181b-2-3p with Downregulated SOX21

The Sox family of transcription factors plays critical roles in the regulation of neurogenesis. SOX1, SOX2 and Sox3 are expressed at high levels in the murine embryonic nervous system [33]. In addition, their involvement in the regulation of neurogenesis has been described extensively [34,35,36]. Similarly, SOX21 may also be involved in neurogenesis in the chick embryos [19] and in adult mice [20]. In the latter study, SOX21 was found to promote neurogenesis in the hippocampus via the transcriptional repression of the Hes5 gene [20]. A significant decrease in the number of cells expressing the SOX1, SOX2 and SOX21 transcription factors in the SGZ of 8-week-old AD transgenic mice further supports their role in neurogenesis. It has been well accepted that the impairment of neurogenesis occurs in the brain of AD patients or animal models [37]. Several stem cell-based therapies as well as stem cell products such as exosomes have shown promising results in the early diagnosis of AD [38]. MUTYH, expressed in the hippocampus of AD and non-AD patients, induced microglial activation with poor neurogenesis, contributing to memory impairment. Its deficiency is highly beneficial for ameliorating AD pathogenesis [39]. These observations are consistent with our result that the overexpression of SOX21 in NSCs blocked the decreased neurogenesis, while the knockdown of SOX21 expression inhibited the activation of microglia induced by γ-irradiation. However, it should be noted that SOX21 may play multiple roles in the regulation of neurogenesis, as a higher concentration of SOX21 inhibits neuron formation and promotes progenitor maintenance based on the study in the Xenopus neural plate [40]. Further studies may still be needed to elucidate the exact role of SOX21 in the regulation of brain neurogenesis.

Our recent study showed that the radiation-induced upregulation of miR-34a-5p and decreased Tia1 in the neonatal mouse brain were involved in hypoplasia and the impairment of neurogenesis in the dentate gyrus, leading to depression in adult mice [3]. The radiation-induced upregulation of miR-181b-2-3p and the downregulation of the SOX21 mRNA and protein in NSCs in the present study suggest that the direct interaction of miR-181b-2-3p with SOX 21 may regulate the radiation-induced impairment of neurogenesis not only by directly targeting NSCs but also by indirectly inducing microglial activation, proliferation and subsequent brain inflammation. The knockdown and overexpression of SOX21 in BV2 and NSCs, respectively, may improve the radiation-induced impairment of neurogenesis.

Based on the finding that the locally irradiated subventricular zone (SVZ) preserved NSCs’ ability to respond to a demyelinating lesion by increasing the number of proliferative cells and neuroblasts, and that the remaining cells in the irradiated SVZ repopulated the neurogenic niche in the absence of damage, Capilla-Gonzalez et al. argued that NSCs were resistant to SVZ radiation [41]. However, in the abovementioned study, radioresistant C57BL/6 mice were used, and the animals were anesthetized with the intraperitoneal injection of ketamine and xylazine prior to the radiation exposure. As ketamine and xylazine have been reported to be neuroprotective [42,43], and increased neurogenesis was observed in the ketamine- and xylazine-anesthetized rat irradiation model [44,45], these results suggested that the experimental findings derived from ketamine- and xylazine-anesthetized animal models may not be comparable with our study involving the radiosensitive awake free-walking Balb/c mouse model. We did observe a slightly higher radioresistance in the neural stem cells compared to that in BV2, as indicated by the increased expression of SOX21 and the decreased levels of miR-181b-2-3p in BV2 cells exposed to 0.5 Gy radiation. However, in NSCs, a SOX21 mRNA change was shown after irradiation with a dose range from 2 to 5 Gy. 

### 4.3. Study Limitations and Conclusions

The present study employed multiple methods—immunohistochemistry, real-time RT-PCR, western blot, ELISA, cell proliferation and cytotoxicity assay—to examine the roles of miR-181b-2-3p and SOX21 in radiation-induced brain inflammation and the impairment of neurogenesis both in vivo and in vitro. Our results suggest that the radiation-induced interaction of miR-181b-2-3p with SOX21 may play dual roles in microglia and NSCs, respectively, leading to the impairment of brain neurogenesis.

Nevertheless, several limitations are associated with our study. In general, small sample sizes were used in the different experiments, depending on the available sample numbers and experimental needs. In order to analyze the small-sized data, the nonparametric Fisher–Pitman permutation test was used instead of parametric statistical methods, because the Fisher–Pitman permutation test is an increasingly attractive alternative to the ANOVA test, and there is no distributional requirement for experimental samples [46]. Moreover, in vitro experiments on BV2 and neural stem cells were conducted separately, and the co-culture of two cell lines may provide more convincing results for understanding the effect of the interaction of miR-181b-2-3p with SOX21 on inflammation and neurogenesis. In addition, the underlying signaling pathways of SOX21 were not examined in this study. It has been reported that Hes5 could act as a downstream effector of Sox21, and Sox21 promoted hippocampal adult neurogenesis through inducing the transcriptional repression of the Hes5 gene [20]. Thus, the functions of SOX21 downstream effectors in γ-irradiation-induced damage should be investigated in future studies. 

## Data Availability

The data presented in this study are available on request from the corresponding author. The data are not publicly available due to ethical restrictions.
